# Obesity as a Risk Factor for Severe COVID-19 in Hospitalized Patients: Epidemiology and Potential Mechanisms

**DOI:** 10.3390/healthcare10101838

**Published:** 2022-09-22

**Authors:** Scarleth Aburto, Mischka Cisterna, Javiera Acuña, Camila Ruíz, Sharon Viscardi, José Luis Márquez, Ines Villano, Pablo Letelier, Neftalí Guzmán

**Affiliations:** 1 Laboratorio de Investigación en Salud de Precisión, Departamento de Procesos Diagnósticos y Evaluación, Facultad de Ciencias de la Salud, Universidad Católica de Temuco, Temuco 4780000, Chile; 2 Núcleo de Investigación en Producción Alimentaria, Universidad Católica de Temuco, Temuco 4780000, Chile; 3 Biotechnology of Functional Foods Laboratory, Camino Sanquilco, Parcela 18, Padre Las Casas 4850000, Chile; 4 Escuela de Kinesiología, Facultad de Ciencias Médicas, Universidad de Santiago de Chile, Santiago 9160000, Chile; 5 Dipartimento di Medicina Sperimentale, Università degli Studi della Campania Luigi Vanvitelli, 81100 Caserta, Italy

**Keywords:** COVID-19, SARS-COV-2, infection, obesity

## Abstract

SARS-CoV-2 infection is a global public health problem, causing significant morbidity and mortality. Evidence shows that obesity is a recognized risk factor for hospitalization, admission to critical care units, and the development of serious complications from COVID-19. This review analyzes the available epidemiological evidence that relates obesity to a higher risk of severity and mortality from COVID-19, examining the possible pathophysiological mechanisms that explain this phenomenon on a cellular and molecular level.

## 1. Introduction

SARS-CoV-2 infection and the disease it produces (COVID-19) constitute an important public health problem. As of July 2022, it has affected 560 million people and caused more than 6.3 million deaths worldwide, and the countries with the highest number of cases are the United States, France, India, Brazil, and Germany [1]. The infection presents a wide range of signs and symptoms, which vary according to the severity of the clinical picture. In mild cases, fever, cough, dyspnea, headache, and myalgia are frequent, while severe cases may present respiratory failure, septic shock, disseminated intravascular coagulation, multiple organ failure, and death [2]. It is estimated that approximately 81% of patients have a mild case, 14% a severe case, and 5% of patients may progress to severe or critical conditions [3]. Despite increasing vaccination rates globally, outbreaks persist in different regions, and patients continue to progress to severe conditions requiring advanced medical care. Studies have described an association between severe forms of COVID-19 and the presence of comorbidities [4]. A recent meta-analysis that included 1576 patients diagnosed with COVID-19 showed that the main comorbidities correspond to hypertension (21%) and diabetes (9.7%), followed by cardiovascular diseases (8.4%) and respiratory system diseases (1.5%) [5]. On the other hand, epidemiological studies show that a high proportion of patients who develop respiratory failure or are admitted to critical care units are obese or overweight, observing a significant association between COVID-19 and BMI [6,7]. Based on the available evidence, the objective of this review is to analyze the effects of obesity as a risk factor in patients with COVID-19, examining the pathophysiological mechanisms that explain this phenomenon.

## 2. Materials and Methods

In this narrative review, a PubMed/MEDLINE search was performed up to May 2022, using Medical Subject Headings (MeSH) selecting studies that were published in peer-reviewed journals. Inclusion criteria for research articles associated with the prevalence of obesity in COVID-19 patients, risk of severe disease or mortality were: (1) Adult study population, over 18 years of age; (2) Diagnosis of COVID-19 confirmed by reverse assay transcriptase Real Time polymerase chain reaction (qRT-PCR) of nasal and pharyngeal swab specimens. Letters to the editor, case reports, editorials, in addition to population studies of subjects < 18 years of age, and patients diagnosed by methods other than molecular detection of SARS-COV-2 were excluded; and (3) Development of the topic.

## 3. Development of The Topic

SARS-CoV-2 corresponds to a positive-sense single-stranded RNA virus that belongs to the Coronaviridae subfamily, which has the capacity to infect mammals and other animals [8]. The viral genome codes for four main structural proteins that correspond to the spike (S), nucleocapsid (N), membrane (M), and envelope E proteins. The S protein is a trimeric glycoprotein that mediates binding to host cells and viral entry. The N protein packages the viral genome in a ribonucleoprotein complex [6]. The M protein shapes the viral envelope and participates in the assembly of virions within the infected cell [2]. Finally, the smallest structural protein corresponds to the E protein, which has important functions in the production, maturation, and assembly of viruses [2,6]. The virus enters the cell through the binding of protein S to angiotensin-converting enzyme type 2 (ACE2), which has two functionally distinct subunits. The S1 surface subunit recognizes and binds to the cell receptor, and the S2 transmembrane subunit facilitates the fusion of the viral membrane with the cell membrane [9].

Obesity is considered one of the main risk factors for developing chronic diseases. On a global level, the age-standardized BMI has steadily increased, while the prevalence of obesity has practically tripled in the last forty years [10,11], especially in women. Reports from the Pan American Health Organization (PAHO) show that the American continent has the highest prevalence of obesity, estimating that it affects 28% of the adult population [12]. Although overnutrition was initially considered a problem that mainly affected high-income countries, a sustained increase has now been observed in low- and middle-income countries [13]. The World Health Organization (WHO) defines overweight and obesity as an abnormal or excessive accumulation of fat that can be detrimental to health. One of the parameters used to identify overnutrition is BMI, which is a simple indicator of the ratio between weight and height, frequently used to identify overweight and obesity in adults. Subjects with a BMI > 25 kg/m^2^ are considered to have overweight and a BMI > 30 kg/m^2^ are considered to have obesity [14]. The classification proposed for obesity distinguishes different degrees of obesity with respect to their weight index: class I obesity (BMI between 30–34.9 kg/m^2^), class II obesity (BMI between 35–39.9 kg/m^2^) and class III obesity (BMI > 40 kg/m^2^) [14].

Evidence shows that obesity is significantly associated with increased severity and mortality in patients with COVID-19 [15,16]. Table 1 shows examples of the frequency of obesity in hospitalized patients in various geographic regions. High frequencies of obesity in patients with COVID-19 are observed in America and Europe, in contrast to those observed in Asian countries. A study of 180 hospitalized subjects with laboratory-confirmed COVID-19 in the United States showed that 48.3% were obese [17], while in France, in a group of 124 critically ill patients, the prevalence of obesity was 46% [18,19,20]. In Latin America, different frequencies of obesity are observed in patients hospitalized for COVID-19, with high frequencies observed in Mexico and Chile [21,22,23,24]. A recent study in 1141 Chilean patients showed that the frequency of obesity was 25.07%. Of these patients, 23.3% presented a serious evolution [25]. The contrast in the frequencies observed in patients with COVID-19 in global studies can be explained, at least in part, by the differences in the prevalence of obesity between populations [26,27,28,29,30,31]. Countries such as the United States, Mexico, and Chile have high obesity rates, ranking among the top 10 countries affected by overnutrition [32]. In addition, epidemiological evidence shows that 18.4% of obese adults are from high-income English-speaking countries, where a higher frequency of severe obesity is also observed (27.1%) [10].

Table 2 presents studies which describe the severity and mortality risk in patients with COVID-19 and obesity, highlighting the clinical relevance of these findings. In general, the accumulated evidence shows that obesity is a risk factor, regardless of other comorbidities, for the development of severe conditions and mortality in patients with COVID-19. A meta-analysis that considers 12 studies (N = 12,591) showed that obese patients show a high risk of developing severe symptoms and requiring invasive mechanical ventilation [33]. A recent meta-analysis and meta-regression in 3,140,413 patients (167 studies) show that obesity was associated with an increased risk of severe disease (RR = 1.52, 95% CI 1.41–1.63, *p* < 0.001) and a high mortality (RR = 1.09, 95% CI 1.02–1.16, *p* = 0.006) [16]. In addition, studies have shown that obesity is associated with a higher risk of mortality among patients with COVID-19 and is higher in patients with class III obesity than in those with class I and II obesity [34]. Finally, by studying a cohort of 1141 cases confirmed by molecular biology, Domínguez et al. [25] showed that obesity is a risk factor for severe disease (critical care and death) (OR 2.36; 95% CI 1.65–3.39), regardless of the effect of diseases, such as diabetes or chronic kidney disease.

## 4. Discussion

Epidemiological evidence shows that between 10–20% of patients with COVID-19 develop a severe case of the disease, presenting major complications, such as acute respiratory distress syndrome, multi-organ failure, and septic shock [36]. Obesity has been recognized as one of the major risk factors for severe COVID-19. A prospective cohort study that evaluated the association between obesity and COVID-19 in 6.9 million people demonstrated a linear increase in the risk of severe disease for hospital admission and death, as well as for admission to critical care units [37]. A study carried out on 120,000 Mexican adults showed that every increase of 5 Kg/m^2^ of BMI increased the risk of mortality by 42%, while individuals with BMI > 40 Kg/m^2^ had a risk of mortality 4 times greater than subjects with a normal weight (<25 kg/m^2^) [38].

**Figure 1:**
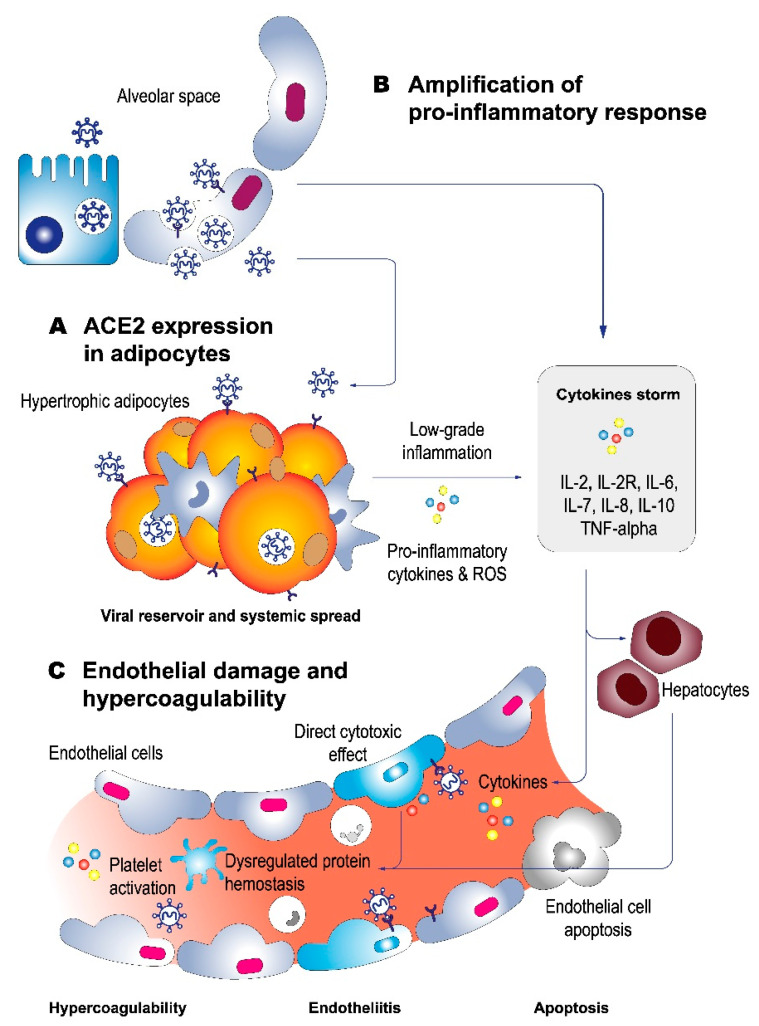
Pathophysiology of complications associated with obesity in COVID-19. Obesity is a recognized risk factor for complications in SARS-Cov-2 infection, which is associated with various mechanisms. SARS-CoV-2 enters the cell through the interaction of the S protein with the ACE2 receptor expressed in various cell types. Proposed mechanisms include: (**A**) ACE2 expression in adipose tissue, which contributes to increased susceptibility to infection and viral systemic spread; (**B**) chronic inflammation and amplification of the pro-inflammatory response, characterized by a deregulation of the immune response associated with progression to severe and critical conditions characterized by multiple organ failure mediated by apoptosis and alteration of lung function, triggering different respiratory complications; and (**C**) endothelial damage and hypercoagulability, a phenomenon mediated by the direct cytotoxic action of the virus on the endothelial cell that expresses ACE2, generating endothelial disease and apoptosis. On the other hand, significant changes have been described in the expression of procoagulant proteins and regulation of fibrinolysis, release of microparticles derived from platelets and platelet activation induced by the generation of reactive oxygen species (ROS), which generates a state of hypercoagulability, predisposing the patient to the development of thrombosis.

Previous evidence shows that obesity has deleterious effects on lung function, which explains its association with lung disease, such as hypoventilation syndrome, obstructive sleep apnea, pulmonary hypertension, and chronic obstructive pulmonary disease, among others [39]. Obesity can affect respiratory mechanics, altering total lung capacity and predisposing obese people to respiratory distress [40]. In addition, abdominal obesity has been shown to restrict movement of the diaphragm and chest wall, resulting in reduced functional residual capacity and hampering mechanical ventilation [41]. By analyzing the post-mortem lung transcriptional profile of obese and non-obese patients with COVID-19, a recent study showed that the expression of 17 genes was associated with BMI. Of these, genes involved in lipid metabolism, insulin signaling, cell cycle, and maturation, such as lymphocyte-specific kinase (LCK), early growth response 2 (EGR2), cyclin-dependent kinase inhibitor 3 (CDKN3), and maternal embryonic leucine zipper kinase (MELK), were positively correlated with BMI [42,43]. Several mechanisms have been proposed to explain the potential ratio between obesity and complications associated with COVID-19 [21], which are presented in Figure 1. Among these mechanisms, the following are noteworthy: (i) greater expression of ACE-2 in adipose tissue, (ii) chronic inflammation and amplification of the pro-inflammatory response, and (iii) endothelial damage and hypercoagulability.

Various mechanisms have been proposed to explain the potential relationship between obesity and complications associated with COVID-19 [44], which are presented in Figure 1. It is widely accepted that the virus enters the host cell through angiotensin-converting enzyme 2 (ACE2), showing that its overexpression can increase infection and viral replication [17,43]. Studies have shown that the expression of this protein in adipocytes is greater than in the lungs and may act as an important viral reservoir. Frühbeck et al. recently demonstrated that obese patients, in addition to expressing ACE2, present overexpression in adipose tissue of various components necessary for viral entry into the cell, such as CD147, DPP4, and NRP1, which would contribute to increasing susceptibility to infection [44]. Animal model studies have shown that a high-fat diet would also generate an overexpression of ACE2 in adipose tissue [45]. Based on this evidence, excess adipose tissue can increase infection and tissue accessibility, favoring viral systemic spread, prolonged virus entry, and excretion [43].

Exacerbated inflammatory response or hyperinflammation is one of the main phenomena associated with the progression to severe cases of COVID-19 [44]. Under physiological conditions, adipose tissue contains immune cells that contribute to the maintenance of adipocyte metabolism and that generate anti-inflammatory cytokine secretion [46,47]. In contrast, obesity is associated with low-grade chronic inflammation, which promotes the development of various chronic diseases. In obese subjects, chronic inflammation added to the increase in proinflammatory cytokines leading to a deregulation of the innate and adaptive immune response, which is associated with greater susceptibility to infections [48]. A key event in the severity of COVID-19 is an uncontrolled immune response known as a cytokine storm [36,48,49], which is associated with progression to severe and critical conditions characterized by multiple organ failure. Apoptosis is a mechanism that relates the cytokine storm with organ damage, demonstrating that various viral proteins of SARS-CoV-2 induce PANoptosis, which involves three pathways of programmed cell death: pyroptosis, apoptosis, and necroptosis. From a clinical standpoint, immune dysregulation leads to an increase in inflammatory markers, such as C-reactive protein, ferritininemia, IL-6, IL-1β, tumor necrosis factor α (TNF-α), and chemokines [6]. In addition, cytokines, such as IL-2, IL-4, IL-10, IFN-γ, and TNF-α present maximum levels in the blood 3 to 6 days after the onset of the disease [50]. Thus, the overload of cytokines produced by the viral infection, added to the low-grade chronic inflammation that obese patients previously present, induces different respiratory complications. Alterations in blood hemostasis have been permanently associated with severe conditions and mortality in COVID-19 [51,52,53]. Alterations in hemostasis markers, such as elevation of D-dimer and prolongation of global coagulation tests (prothrombin time and activated partial thromboplastin time), have been described in severely ill patients [54]. Various mechanisms could explain, at least in part, the hypercoagulability observed in obesity and its relationship with severity and mortality associated with COVID-19. First, recent evidence has shown the expression of ACE-2 in the endothelium of various organs, meaning that endothelial cells are essential in the initiation and spread of severe COVID-19 [55]. Post-mortem histopathological analyses have shown the presence of viral elements in endothelial cells with an accumulation of inflammatory cells, suggesting that SARS-CoV-2 infection induces endotheliitis, apoptosis, and pyroptosis, an important mechanism in endothelial injury in patients with COVID-19 [56]. Second, the elevation of inflammatory cytokines by adipose tissue induces changes in hemostasis proteins, generating a tendency toward hypercoagulability. Obese patients present an increased expression of procoagulant factors and proteins that regulate fibrinolysis, such as plasminogen activator inhibitor (PAI-1). In addition, an increase in circulating platelet-derived microparticles and deregulation of fibrinolytic markers in obese patients have been previously reported, positively correlated with BMI and excess adipose tissue, which generates platelet activation and dysregulation of hemostasis [57]. Based on this, the increase in inflammatory cytokines in COVID-19, including IL-1α expressed in platelets, monocytes, and endothelial cells under proinflammatory conditions, constitutes a link between the inflammatory response and activation of the coagulation system [58]. Finally, obesity is characterized by the presence of oxidative stress, which induces platelet dysfunction. Along these lines, some authors propose a potential mechanism related to the production of reactive oxygen species (ROS), with consequent activation of platelets and generation of thrombin [59], which triggers a state of hypercoagulability with a greater tendency toward thrombotic phenomena.

As we have mentioned before, comorbidities are important risk factors in the development of a severe or fatal COVID-19 syndrome. The relevance of understanding obesity as one of the most important risk factors was discussed, however, other conditions and comorbidities related to poor health, such as advanced age, diabetes, and hypertension are risk factors for severe and fatal courses of diseases [60]. This is associated with organ damage, mainly affecting the heart, liver, and kidneys [61,62,63]. To understand the importance of risks factors could help to predict patients’ outcomes and in this context multivariate regression indicated age over 65 years (*p* < 0.001), smoking (*p* = 0.001), critical disease status (*p* = 0.002), diabetes (*p* = 0.025), high hypersensitive troponin I (>0.04 pg/mL, *p* = 0.02), leukocytosis (>10 × 109/L, *p* < 0.001), and neutrophilia (>75 × 109/L, *p* < 0.001) predicted unfavorable clinical outcomes. In contrast, the administration of hypnotics was significantly associated with favorable outcomes (*p* < 0.001) [61]. It is also reported that older age, male, fever over 38.5 °C, symptoms of dyspnea, pneumonia, and underlying comorbidity are the risk factors most associated with severity of disease [62,63]. 

## 5. Conclusions

Based on the evidence accumulated in epidemiological studies that analyze the ratio between overnutrition and COVID-19, obesity constitutes a recognized risk factor for severity and mortality in individuals infected with SARS-CoV-2 and is closely associated with complications of the disease. Various pathophysiological mechanisms explain the development of complications in obese patients, including increased expression of ACE2 in adipose tissue, chronic inflammation, and amplification of the pro-inflammatory response, in addition to endothelial damage and hypercoagulability. The understanding of the mechanisms and the effect of adipose tissue on the predisposition to severe conditions of the disease suggest that the management of obesity could contribute to a reduction in the morbidity and mortality of SARS-CoV-2 infection, especially in countries with high rates of overnutrition. Likewise, based on this scientific evidence, obese patients who are hospitalized for COVID-19 must be monitored, using laboratory biomarkers that enable early detection of progression to severe disease.

## Figures and Tables

**Table 1:** Frequency of obesity in patients hospitalized for COVID-19 in various geographic regions.

Continent	Country	Total Patients, *N*	Obesity Frequency (%)	Reference
North America	United States	180	48.3	[17]
	Mexico	100	29	[20]
		51,633	20.7	[21]
		3844	17.4	[22]
South America	Chile	169	33	[23]
		47	44.7	[24]
		1141	25.07	[25]
	Brazil	1152	18.9	[26]
Asia	Japan	580	12.1	[27]
	China	1091	26.2	[28]
		297	13.47	[29]
Europe	France	124	46	[18]
	Italy	92	31.5	[30]
		482	21.6	[31]

**Table 2:** Risk of severity and mortality in obese patients with COVID-19.

No. Cases	Risk (Risk; 95% CI; *p*)	Clinical Relevance	Reference
12,591 Meta-analysis	Obesity was associated with a 1.79 times higher risk of developing poor outcomes of COVID-19 (OR 1.87; 95% CI 1.55–2.26; *p* < 0.00001). Obesity was associated with increased need for ICU intervention (OR 1.57; 95% CI 1.18–2.09; *p* = 0.002) Obesity was associated with a higher risk of COVID-19 disease progression (OR 1.41; 95% CI 1.26–1.58; *p* < 0.00001).	Increased risk of severe COVID-19 and increased demand for ICU care in patients with obesity.	[33]
543,399 Meta-analysis	Significantly increased risk of mortality with obesity (RR 1.42; 95% CI 1.24–1.63, *p* < 0.001) Class III obesity was strongly associated with an increased risk of mortality (RR 1.92; 95% CI: 1.50–2.47, *p* < 0.001).	Obesity is associated with an increased risk of mortality in patients with COVID-19. The risk of mortality is higher in patients with class III obesity.	[34]
482	BMI between 30–34.9 kg/m^2^ significantly increased the risk of respiratory failure (OR 2.32; 95% CI: 1.31–4.09, *p* = 0.004) and admission to the ICU (OR 4.96; 95% CI 2.53–9.74, *p* < 0.001). Higher risk of death was observed in patients with a BMI ≥ 35 kg/m^2^ (OR 12.1; 95% CI 3.25–45.1, *p* < 0.001).	Obesity is a strong, independent risk factor for respiratory failure, admission to the ICU and death among COVID-19 patients. A BMI ≥ 30 kg/m^2^ identifies a population of patients at high risk for severe illness, whereas a BMI ≥ 35 kg/m^2^ dramatically increases the risk of death.	[31]
297	Overweight (OR 4.222; 95% CI 1.322–13.476; *p* = 0.015) and obesity (OR 9.216; 95% CI 2.581–32.903; *p* = 0.001) were independent risk factors of severe illness. Obesity (OR 6.607; 95% CI 1.955–22.329; *p* = 0.002) was an independent risk factor of respiratory failure.	Overweight and obesity were independent risk factors of severe illness in COVID-19 patients.	[29]
3615	Patients aged < 60 years with a BMI between 30–34 kg/m^2^ present an increased risk of acute admission (OR 2.0; 95% CI 1.6–2.6; *p* < 0.0001) and critical care (OR 1.8; 95% CI 1.2–2.7; *p* = 0.006). Patients with a BMI ≥ 35 kg/m^2^ and aged < 60 years present an increased risk of acute admission (OR 2.2; 95% CI 1.7–2.9; *p* < 0.0001) and critical care (OR 3.6; 95% CI 2.5–5.3; *p* < 0.0001).	Obesity appears to be a previously unrecognized risk factor for hospitalization and ICU needs.	[35]

OR = Odds Ratio; RR = Relative risk.
